# People with Diabetes Mellitus: Soft target for COVID-19 infection

**DOI:** 10.12669/pjms.36.COVID19-S4.2629

**Published:** 2020-05

**Authors:** Arshad Hussain, Iftikhar Ali, Zair Hassan

**Affiliations:** 1 Dr. Arshad Hussain, FRCP (Edinburgh). Department of Medicine & Allied, Northwest General Hospital & Research Centre, Peshawar, Pakistan; 2 Iftikhar Ali, PharmD, M.Phil., MPH. Pharmacy Unit, Paraplegic Center, Hayatabad, Peshawar, Pakistan; 3 Dr. Zair Hassan, MBBS. Department of Medicine, Hayatabad Medical Complex, Peshawar, Pakistan

**Keywords:** COVID-19, Diabetes Mellitus, Viral infection

In December 2019, in the Hubei province (China), no one could have predicted the pandemic potential of the Severe Acute Respiratory Syndrome Coronavirus 2 (SARS-CoV2 or COVID-19).^1^ It’s almost two months since the World Health Organization (WHO) has declared it as a global pandemic.^2^ The outbreak has caused concerns for health care systems across the globe and mounted fear and anxiety as the situation continue to worsen. Surprisingly the number of cases reported in South East Asia still remains relatively low as compared to Europe. However, there are other dilemmas facing these countries.

An estimated, 5-15% of the world’s population contract influenza every year with estimated 290 000 to 650 000 respiratory deaths.^3^,^4^ The figure for China is around eighty-eight thousand persons annually in excess of any potential outcomes of COVID-19.^5^ Diabetes mellitus (DM) itself is an important risk factor for various adverse outcomes. So the fear is of worst outcomes rather than just simply contracting the disease. Mortality rate in Hong Kong in ≥75 age group with pneumonia surpasses the mortality rate in this age category from cardiovascular disease and cancer.^6^ Generally when people with DM are infected with a virus, they tend to have more severe symptoms and with more complications. The data is still emerging regarding DM and COVID-19. The risk in diabetic patient has been likewise reported in the previous CoV infections, SARS in 2002 affecting more than eight thousand in Asia and the Middle East^7^,^8^ and two thousand in Middle East respiratory syndrome (MERS) in Saudi Arabia in 2012.^9^

It is widely accepted that diabetics have an increased propensity to develop infections.^10^ Comorbid conditions such as cardiovascular disease, DM, hypertension and obesity have substantial effect on outcome of a patient infected with COVID-19.^11^,^12^ The large DM population across the globe makes this concern of keen interest as the pandemic progress. However, how COVID-19 affects diabetics more likely, the prognosis is not yet understood. As of this time emerging data mostly from China, shows that diabetics are more prone to get COVID-19 infection. A study by Guan W, et al,^13^ reported that out of 1099 COVID-19 patients, 173 with severe disease existed the comorbidities such as DM (16·2%), hypertension (23·7%), coronary heart diseases (5·8%), and cerebrovascular disease (2·3%). In another study from Wuhan by Zhang JJ, et al,^14^ of 140 COVID-19 hospitalized patients, 30% had hypertension and 12% had DM. A meta-analysis by Li B, et al, comprising 1527 patients with COVID-19 stated 9.7% prevalence of diabetes. Notably, the presence of diabetes was correlated with a two-fold greater risk of severe disease or necessitating intensive care treatment, signifying prognostic impact of this comorbidity.^15^ Similarly, Fang L et al., suggested that patients with diabetes, hypertension or other comorbidities, who have used ACEI (angiotensin-converting enzyme inhibitors), are more prone to contract COVID-19 infection.^11^ A high case fatality rate (73.3%) has been observed in COVID-19 patients with underlying diseases.^13^ In patient with these comorbidities, potential factors might be due to direct accelerated damage of target tissues or favoured virus life-cycle during a SARS-CoV-2 infection.

The emerging data also portrays high risk for complication including death in these patients. A study by Yang X, et al.^12^ described, that DM was the most distinctive comorbid condition of non-survivors. A recent study by Chinese Center for Disease Control reported the overall summary of all COVID-19 cases (72,314) which showed 7.3% case fatality in DM.^16^ These findings are in line with published data in those with respiratory infection.^7^-^9^

Generally, the increased frequency of infections among diabetics seems to be associated with hyperglycemia, while these infections may increase the morbidity; they may also be the first manifestation of DM or a precipitating factor for complications. Till now, there are few studies that investigated the role of elevated blood glucose level in the pathophysiology and outcome in respiratory viral disease.^6^-^8^,^16^ But it has been described that high blood glucose can increase glucose concentrations in airway secretion.^9^ In vitro pulmonary epithelial cells exposure to high glucose levels lead to an increased influenza viral infection and replication, thus predicting same in vivo. These results are in agreement with the article published on DM patients infected with avian influenza whereby hyperglycemia was associated with a poor outcome.^17^ With coexistent diabetes and viral respiratory diseases (COVID-19) tight glycemic control has beneficial clinical outcome. People, whose diabetes is not well managed, tend to develop complications of diabetes much earlier.

Diabetes coexists with hypertension most of the time. Hypertensive patients with diabetes are treated most of the time with ACEI and angiotensin Type-I receptor blockers (ARBs) drugs. A unique observation is postulated that coronaviruses bind to angiotensin converting enzyme 2 (ACE2) suggesting that patient treated with (ACEI and ARBs) lead to elevated levels of ACE2 placing diabetics at higher risk.^11^ Studies have also showed that high expression of ACE2 in these patients might facilitate SARS-CoV-2 to enter the targeted cells in the respiratory system, and prolong the time of viral clearance.^11^ SARS-CoV-2 viral clearance is the gold standard for describing the recovery of COVID-19 infections.

The difference in rates and severity of infection in Type-I versus Type-II DM patients and in male versus female patient is still unclear. However, it is obvious that Sex, age and immunity are prominent biological factors during combating infectious pathogens.^18^ Females may have lower susceptibility to viral infections, because estrogen and progesterone can help to increase the innate and adaptive immune responses, and many immune genes are X-linked. High immune reactivity post viral infection in women can accelerate the process of viral clearance.^19^,^20^

What we know from China, that diabetics who contracted the disease had more serious complications and relatively more deaths than the general population. All this requires a careful management of people with diabetes; they need to be closely monitored with regular checks on the blood glucose levels. The threshold for COVID-19 testing in people with diabetes needs to be lower than the general population as well as a lower threshold for hospitalization in these patients. To minimize contact and enforce the principle of social distancing many diabetic patients are cancelling their routine visits, this along with limited chances of mobility and exercise and a constant stress of being forced to stay at home, means worsening of the blood glucose levels and increased risk of adverse events. All these factors play a major role in enhancing their vulnerability to COVID-19 infections. Adherence to WHO/Local guidelines regarding social isolation In patient with these comorbidities, potential factors might be due to direct accelerated damage of target tissues or favoured virus life-cycle during a SARS-CoV-2 infection. Finally, the current situation emphasizes the need for more clinical investigation as the pandemic unfolds to fully characterize the problem and define best practices for optimum outcomes. It’s also important that people with diabetes follow sick day rule. Sick day rule includes, staying hydrated, monitoring blood sugars frequently, monitoring temperature, if on insulin, to checking ketones in blood and following healthcare team recommendations. At home family members should take all precautions and follow the local guidelines in keeping places around very clean and may be a protracted space be made available if possible for the vulnerable household members like elderly members with underlying health conditions like diabetes.

## Authors’ Contribution

**AH:** Conception, Drafting of initial version.

**IA:** Addition of contents to the initial version, organization, review & editing.

**ZH:** Added ideas and contents to the first version and language editing.

All authors read and approved the final version.
